# Cross-cultural adaptation and test-retest reliability assessment of a Swedish version of the exercise adherence rating scale in patients after shoulder surgery

**DOI:** 10.1080/07853890.2024.2409962

**Published:** 2024-10-01

**Authors:** Johanna Söderberg, Ellen Sallfeldt, Eva Ribom, Charlotte Urell

**Affiliations:** a Department of Surgical Sciences, Orthopedics, Uppsala University, Uppsala, Sweden; b Department of Women’s and Children’s Health, Physiotherapy, Uppsala University, Uppsala, Sweden

**Keywords:** Exercise therapy, Swedish, psychometric, questionnaire, shoulder

## Abstract

**Background:**

Adherence to prescribed home exercise is an important predictor for the long-term effectiveness of exercise therapy and therefore important to evaluate. The Exercise Adherence Rating Scale (EARS) is a valid and reliable tool to assess exercise adherence behavior, but it is not translated into Swedish. This study aimed to translate EARS into Swedish and to explore the psychometric properties in terms of test-retest reliability, internal consistency as well and possible floor-/ceiling effects.

**Materials and Methods:**

A translation and cultural adaptation process followed international guidelines and resulted in EARS-Sv. A total of 30 patients who had undergone shoulder surgery were included in the study and filled out EARS-Sv at two different time points. The test-retest reliability was evaluated through the weighted kappa coefficient and Intraclass Correlation Coefficient (ICC). Cronbach’s alpha was used to assess internal consistency. Floor-/ceiling effects were calculated.

**Results:**

The test–retest reliability of the questionnaire was good with ICC (0.79, CI 95%) and moderate with weighted kappa-coefficient (MD= 0.58). Cronbach’s alpha was considered good (0.88). A ceiling effect was registered in all 6 items of EARS-Sv.

**Conclusion:**

EARS-Sv has moderate to good test-retest reliability and good internal consistency in patients who have undergone shoulder surgery.

## KEY MESSAGES

The postoperative results in orthopaedic patients are often dependent on adherence to postoperative home rehabilitation programs.

Exercise Adherence Rating Scale (EARS) is a questionnaire assessing adherence to prescribed home exercise and the Swedish version has an acceptable test-retest reliability and good internal consistency for patients who have undergone shoulder surgery.

The Swedish version of EARS could be used as a tool to identify patients having difficulties adhering to the prescribed home exercises.

## Introduction

Home exercise programs can be a beneficial way to reduce pain and improve physical function in various conditions [1–3]. The postoperative results in ortho­pedic patients are often dependent on fulfilling postoperative home rehabilitation programs [4–6] and adherence to these programs is an important predictor for the long-term effectiveness of the exercise therapy [7]. However, patient adherence to home exercise programs is relatively poor [8]. In a long-term perspective, it is not only the function and quality of life of the individual that is negatively affected by low adherence but also increased societal costs [9].  

Adherence has been defined by the World Health Organization [9] as to which extent a person’s behavior corresponds to the recommendations of a health care professional. Compared to compliance, adherence refers to a more active involvement of the patient in the planning and implementation of the treatment [9,10]. Several factors can be related to a patient’s adherence to home physiotherapy, where self-efficacy emerges as one of the most important [11]. Adherence to non-pharmacological treatment is measured by different methods [12,13]. Frost et al. [12] analyzed eight different measurement methods used to evaluate home rehabilitation and concluded that there is no criterion standard of adherence measurement for home therapies.

In 2017, Newman-Beinart et al. [14] developed a valid and reliable questionnaire assessing adherence to prescribed home exercise, the Exercise Adherence Rating Scale (EARS). The EARS is available in English [14], Nepalese [15], Japanese [16], Brazilian Portuguese [17] and Danish [18]. Recently, EARS has been used as a primary tool to assess adherence to home exercise programs in individuals with knee osteoarthritis [19–21] as well as individuals with prediabetes and diabetes [22]. If a physiotherapist can use a self-reported questionnaire on adherence to home exercise to identify areas and behaviors that constitute barriers or facilitators, individualized guidance of the patient can be facilitated.

To our knowledge, there is no valid scale in Swedish to assess adherence to prescribed home exercise, either in a general population or in orthopedic patients. Therefore, the objectives of this study were to translate and adopt EARS into Swedish and to explore the psychometric properties of EARS in terms of test-retest reliability, internal consistency as well as possible floor/ceiling effects in patients who had undergone shoulder surgery.

## Materials and methods

The development and reliability assessment of the Swedish version of EARS (EARS-Sv) consisted of two steps described further on in procedures. Briefly, the first step included the translation and cross-cultural adaptation of EARS to a Swedish version. This was achieved by following established international guidelines [23,24]. The second step was to evaluate the test-retest reliability, internal consistency and floor-/ceiling effects of the EARS-Sv. Before starting the translation procedure, permission for the cross-cultural adaptation of the questionnaire from English to Swedish was obtained from the developer of EARS [14]. The study was approved by the Swedish Ethical Review Authority (D-2019-03995) and the procedures followed the Helsinki Declaration. Written informed consent was obtained from all participants.

### Participants

Patients that had undergone shoulder surgery at Uppsala University Hospital and were referred to the physio­therapy outpatient clinic for postoperative rehabilitation, between April 2018 and September 2020, were recruited. Thirty-four consecutive patients were informed about the purpose and measurement procedures of the study and asked on a voluntary basis to participate. Thirty-three accepted and gave their informed consent. Inclusion criteria were (1) age over 18 years, (2) had shoulder surgery for a large cuff or small cuff rupture (classified by an orthopedic surgent based on the quality of the muscle, degenerative status, retraction of the tendons and the accumulation of fat in the muscle), anterior or posterior instability, arthritis or another injury (3) ability to read and write in Swedish. The exclusion criteria were cognitive impairment.

### Measurement

Exercise Adherence Rating Scale (EARS) consists of 6 items evaluating adherence to prescribed home exercise (Table 1). Examples of inquiries in EARS are: ‘I do my exercises as often as recommended’, ‘I forget to do my exercises’ and ‘I do less exercise than recommended by my healthcare professional.’ EARS utilizes a five-point Likert scale (0-completely agree, 4-completely disagree) for each item, with a total possible score ranging from 0 to 24, where higher scores indicate greater adherence. Items 1,4 and 6 are reversely scored. The original study by Newman-Beinart et al. [14] examined the validity and reliability of EARS among a sample of 224 patients suffering from chronic low back pain. The construct validity of the EARS questionnaire was evaluated using exploratory categorical data factor analysis (EFA) and revealed the presence of one factor loading with an eigenvalue exceeding 1. All 6 items loaded strongly on the factor. Furthermore, test-retest reliability analysis of EARS showed good internal consistency (Cronbach’s alpha= 0.81) and high test-retest reliability (ICC = 0.97) [14]. These findings were replicated in both Brazilian [17] and Nepalese [15] studies. Background data, including age, sex, type of surgical procedure, and time from surgery, were collected from participants.

**Table 1.** Exercise adherence Rating scale (EARS). Items that are rated using a Likert scale (0-4, completely agree to completely disagree).

I do my exercises as often as recommended. I forget to do my exercises. I do less exercise than recommended by my healthcare professional. I fit my exercises into my regular routine. I don’t get around to doing my exercises. I do most, or all, of my exercise.

### Procedure

**Figure 1.**
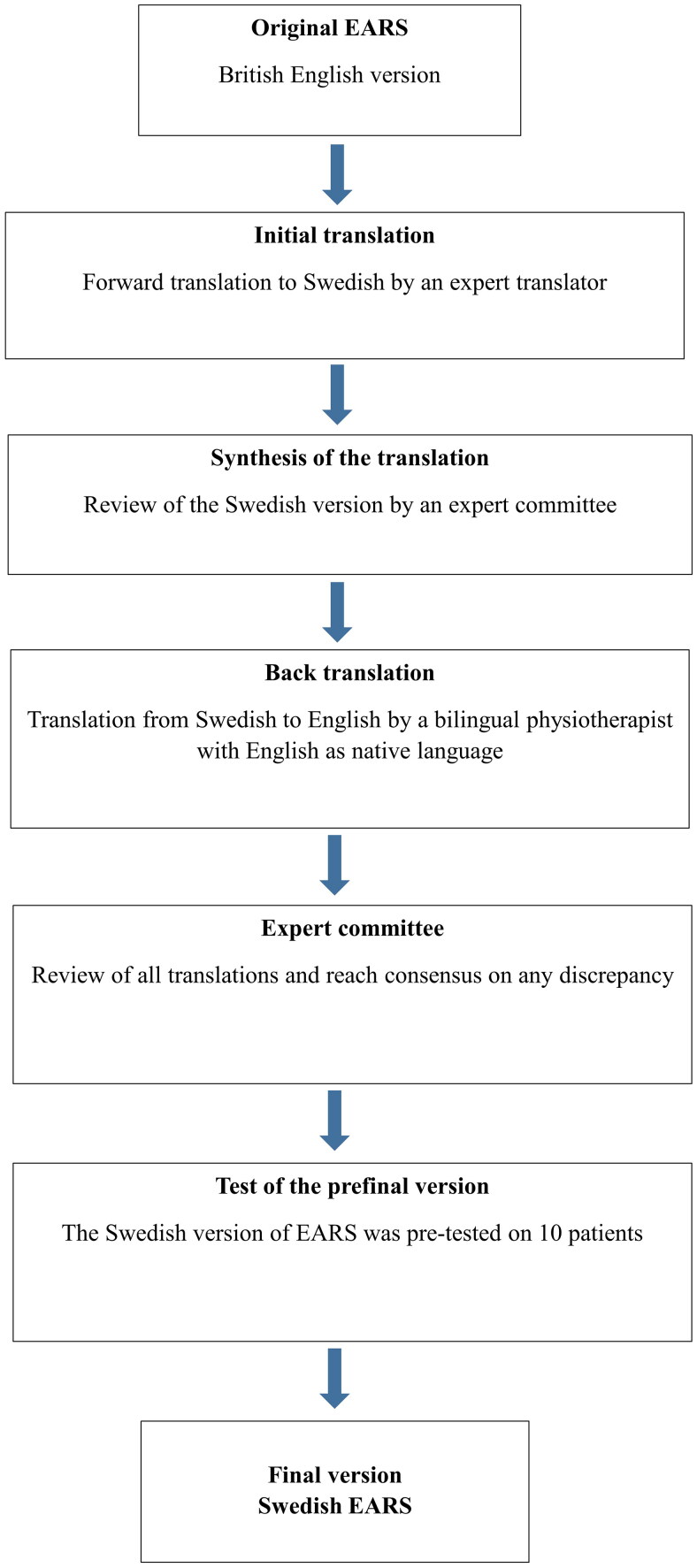
Flow-chart over the development of the Swedish version of EARS.

In step 1, the adaptation of EARS followed established guidelines [23,24]. An independent professional translator translated the original EARS into Swedish. An expert committee, consisting of three physiotherapists with great expertise and knowledge of orthopedic physiotherapy both in clinic and research, examined the Swedish version. A fourth independent physiotherapist with English as a native language did the translation back to English. The expert committee analyzed the two English versions and reached a consensus on a few minor discrepancies. A pre-testing of the preliminary Swedish EARS was conducted on a group of 10 patients from a rehabilitation group after knee surgery. The patients were asked to comment on the comprehensibility of the questions and to give recommendations for improvement. The cross-cultural adaption process including translation is summarized in Figure 1.

In step 2, the inclusion of participants was conducted by four physiotherapists at the physiotherapy orthopedic outpatient clinic at Uppsala University Hospital. During the standard follow-up visit with the physiotherapist at 8 to 12 weeks after surgery, the patients were asked to complete EARS-Sv. Shoulder rehabilitation for each patient was based on standardized rehabilitation plans which included home exercise. The exercises were individually adapted and intensified according to the different healing stages and the patient’s rehabilitation goals. The rehabilitation plans included repeated follow-ups with a physiotherapist [25]. The first round of EARS- Sv was set at 8 to 12 weeks after surgery based on relative clinical stability. The second round of the questionnaire was conducted again after 2-4 weeks, which is a typical interval in this type of study [26].

### Statistical analysis

All statistical calculations were performed using R version 4.0.3 [27]. Demographic data including age, number of weeks from surgery to first time completion of EARS-Sv and the result for EARS-Sv are presented as mean and ± SD. Sex and type of surgery are presented as absolute numbers and proportions, % (Table 2). Reliability was calculated using weighted kappa analysis. The strength of the kappa was determined according to Landis and Koch [28] 0–0.20 as slight, 0.21–0.40 as fair, 0.41–0.60 as moderate, 0.61–0.80 as substantial, and 0.81–1 as almost perfect agreement. Absolute agreement for each item was shown in percentage. A result of 75% or higher was an acceptable level of agreement [29]. To measure the agreement for the total score of EARS-Sv the intraclass correlation co­efficients (ICC), by 2 way mixed-effects model, single measurement, absolute agreement, and its confidence interval were calculated [30]. The ICC was interpreted according to Koo and Li [30] where values less than 0.5 are indicative of poor reliability, values between 0.5 and 0.75 indicate moderate reliability, values between 0.75 and 0.9 indicate good reliability and values greater than 0.90 indicate excellent reliability. The error of measurement was expressed by the standard measurement error (SEM) using the formula SEM = SD√1-ICC, in which SD = standard deviation [31]. A Bland-Altman plot was also included showing the average score between the two time points versus the difference between the two time points with the limit ± 1.96 multiplied by the standard deviation of the difference [32]. Internal consistency was measured by Cronbach’s alpha. Equal or > 0.7 was considered acceptable results [26]. Floor-ceiling effects were also examined. If 15% or more had the highest or lowest score, a floor or ceiling effect was attained [33].

**Table 2.** Characteristics of participants, *n* = 30.

Variables	Baseline values *n* = 30
Age, years (mean ± SD)	57 ± 14
Women, n (%)	5 (17)
Surgery	
Large cuff rupture, n (%)	9 (30)
Small cuff rupture, n (%)	6 (20)
Stabilizing surgery, n (%)	5 (17)
Prosthesis, n (%)	5 (17)
Other, n (%)	5 (17)
Number of weeks from surgery (mean ± SD)	9 ± 1
EARS-Sv. 0-24 points (mean ± SD)	20 ± 5
Prior shoulder surgery, n (%)	8 (27)
Prior rehabilitation recommended by a physiotherapist, n(%)	18 (60)

Higher score in EARS-Sv indicating greater adherence.

Other: pectoralis major tendon repair, distal clavicle fracture fixation, SLAP-tear repair and subacromial decompression.

## Results

### Step 1: cross-cultural adaptation

After translation to Swedish by a professional tran­slator a few modifications were made to the Swedish version of EARS in consultation with the expert committee to secure the cultural adaptation and the comprehensibility of the items. A pretesting of the modified Swedish version of EARS was done on 10 patients, who were not included in the reliability testing. The patients left remarks about the comprehensibility of the questionnaire. One optioned for a reversed scale: ‘completely disagree = 0, completely agree = 4’ and one emphasized the disadvantage of mixing positive and negative questions. No modifications to the Swedish version were made after consulting with the expert committee since these kinds of modifications would have altered the composition of the questionnaire to too great of an extent. The result was EARS-Sv, Supplementary Appendix A.

### Step 2: test- retest reliability assessment of EARS-Sv

Of the 33 patients recruited, 3 patients were excluded; one patient declined participation after the first round, one patient repeatedly answered 0 in all items which was interpreted as incomplete answers and one patient did not complete the second round of the questionnaire. The responses of the 30 participants who completed both rounds were analyzed. The characteristics of the participants including age, sex, type of surgery, EARS-Sv and prior rehabilitation are shown in Table 2. Weighted kappa for each item of EARS- Sv showed fair to substantial correlation with values variating between 0.33-0.64 (MD = 0.58). The absolute agreement for each item varied between 63% and 77% (Table 3).

**Table 3.** Weighted kappa coefficient and absolute agreement for each item in EARS-Sv. n = 30.

EARS item	Weighted kappa	95 % CI	Absolut agreement (%)
1	0.56	0.36–0.77	67
2	0.59	0.35–0.82	63
3	0.60	0.37–0.84	73
4	0.64	0.45–0.84	73
5	0.52	0.24–0.80	77
6	0.33	−0.01-0.67	73

CI: confidence interval.

EARS item: 1. I do my exercises as often as recommended, 2. I forget to do my exercises, 3. I do less exercise than recommended by my healthcare professional, 4. I fit my exercises into my regular routine, 5. I don’t get around to doing my exercises, 6. I do most, or all, of my exercise.

**Figure 2.**
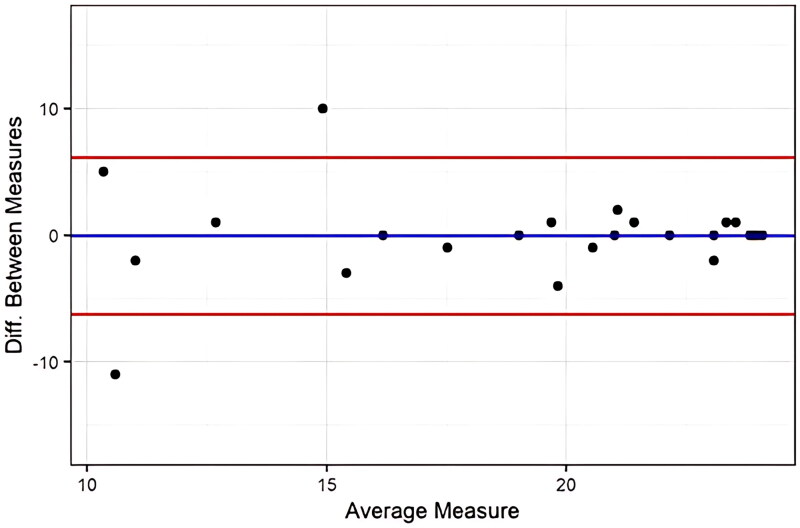
Bland-Altman plot for EARS-Sv describing agreement between two measurements, with an interval of 2-4 weeks between the two test points.

ICC for the total score of EARS-Sv was 0.79 (95% CI 0.60–0.89) which indicates good reliability. The SEM was 2.25 for the total score of EARS-Sv and 0–24 were the minimum and maximum detectable values. The Bland-Altman did not show any systematic bias. There were two outliers and they were not extreme (Figure 2). The Cronbach’s alfa was 0.88 (CI:0.82–0.95) indicating good internal consistency. There was an absence of floor effect. However, ceiling effect was noticed in all 6 items of EARS-Sv.

## Discussion

This study shows that a Swedish version of EARS (EARS-Sv) has good comprehension among an expert committee and a pilot group indicating a good cross-cultural adaptation. It also has a moderate to good test-retest reliability measured by weighted kappa and ICC. Additionally, this study showed good internal consistency, a ceiling effect was noticed in all 6 items of EARS-Sv.

To ensure the comparability of the original EARS and EARS-Sv, the translation and cultural adaptation was carried out according to established guidelines [23,24]. No content or language-related issues were revealed during the translation steps or the adaptation procedure. This corresponds to the Nepalese [15] and Danish [18] studies.

Regarding reliability, weighted kappa was included as a measurement of reliability for each item separately and showed a fair to substantial kappa for EARS-Sv. However, comparisons cannot be made as no other studies assessing EARS have used kappa as a measurement of reliability [14–18]. As the level of adherence is estimated by the total score of the 6 items of EARS, it can be disputed that the ICC is a more suitable analysis method for this type of questionnaire as it evaluates the reliability of the total score. The ICC of EARS-Sv was considered good (0.79, CI 0.60–0.89). Though the results are lower than in the original study (0.97, CI 0.94–0.98) [14] and the Brazilian study (0.91, CI 0.86–0.94) [17], the ICC value of EARS-Sv is still within acceptable limits [33]. The SEM for the total score of EARS-Sv was 2.25 which is in line with the Brazilian study (SEM 1.97) [17]. The original study by Newman-Beinart et al. [14] has not described SEM. A Bland-Altman plot was added to highlight any systematic bias or extreme outliers. The results of the plot were satisfying, showed no indication of bias, and identified only a few outliers who were not extreme outliers. In contrast to other studies evaluating EARS, this study is the only study with participants in a postoperative state [14–18]. Postoperative conditions are changeable over time. Between the two test rounds, changes may have occurred to the patients’ well-being which may have influenced the responses on the second round of EARS-Sv. This could affect the ICC value negatively. To minimize the risk of a change over time, the test-retest was conducted 8–12 weeks after surgery when the operation wound was healed, and the acute postoperative pain had decreased.

The internal consistency of EARS-Sv was considered good, which corresponds with the results of the original [14] and Brazilian [17] versions of the scale but is somewhat lower than the results in the Nepalese study [15].

A ceiling effect was detected in all items of EARS-Sv limiting the ability of the questionnaire to detect change over time and making it difficult to distinguish individuals from each other [33]. A substantial ceiling effect was also detected in the Japanese study [16] but was not evaluated in the original study [14]. The voluntary participation in this study may have resulted in patients with high motivation and good adherence to their rehabilitation failing to give a nuanced description of the postoperative population. There is also a risk of participants overestimating their behavior and giving answers that are perceived as acceptable rather than accurate. Prior studies have shown that information and prior experience of rehabilitation have a positive impact on adherence behavior [9,34]. The fact that most of the participants in this study had had experience with rehabilitation may have influenced the high scores of EARS-Sv. Additionally, the physiotherapists used the written patient information as support when including the participants. This information about adherence to home exercise programs and its impact on the rehabilitation outcome could also have had a positive impact on the participant's adherence and contributed to the ceiling effect of EARS-Sv.

Rotator cuff pathology is a common cause of shoulder pain and is correlated with age. The average age for a unilateral tear is 58.7 years [35]. This corresponds well with the sample in this study where rotator cuff rupture was the leading cause of surgery (*n* = 15), and the mean age of the participants was 57 years. The sample included both sexes, however, the distribution between women (*n* = 5) and men (*n* = 25) should be considered before generalizing the result among a female population. The sample size of this study was small (*n* = 30) but within the recommendation of sample size in reliability studies with continuous data [36].

In addition to EARS, 2 other sections can be used to better understand the adherence behavior; Section A: Prescribed exercise questionnaire and Section C: What helps or hinders doing your exercises. Section C consists of 10 questions regarding the reasons for adherence or non-adherence. In the original study [14], Section C was not included in the final 6-items EARS questionnaire due to low baseline values. In a clinical setting, Section C could be used as a complementary tool in understanding patient’s adherence or lack of adherence to prescribed home exercise. As in the main study [14], Section C was not analyzed in this study. This could be a limitation in the understanding of rehabilitation-specific and personal factors that contribute to home program adherence.

In the orthopedic field, adherence to rehabilitation is essential to achieve the best possible outcome after surgery. Some patients or groups of patients may need more organized rehabilitation in order to adhere to the prescribed home exercises. For optimal results after orthopedic surgery, EARS-Sv could be used as a tool to identify these patients.

According to COSMIN (Consensus-based Standards for the Selection of health Measurement Instruments), an instruments quality should be examined by assessing reliability, validity, responsiveness, and interpretability [37]. In the future it could therefore be of value to continue the assessment of other psychometric properties of EARS-Sv. It should be noted that other studies have shown acceptable construct and structural validity of EARS [14,15,17].

## Conclusions

EARS has successfully been cross-culturally adapted to Swedish. This study shows acceptable test-retest reliability and good internal consistency for EARS-Sv for patients who had undergone shoulder surgery. However, it should be noted that the questionnaire has limitations such as ceiling effect in all 6 items. In further studies, it would be interesting to examine other psychometric properties of EARS-Sv and on a wider population.

## Supplementary Material

Appendix_A_.docx

## Data Availability

Data are available upon reasonable request from the corresponding author.
