# Supplementary material for: Cross-cultural adaptation and test-retest reliability assessment of a Swedish version of the exercise adherence rating scale in patients after shoulder surgery
Source: Ann Med. 2024 Oct 1;56(1):2409962. doi: 10.1080/07853890.2024.2409962 (PMC11445904; doi:10.1080/07853890.2024.2409962)
Supplement: Appendix_A_.docx [file IANN_A_2409962_SM5292.docx]

**Appendix A**

**Del B: Självskattningsskala för hur man följer sitt träningsprogram**.

Här följer sex påståenden. Kryssa i den ruta som bäst beskriver hur du gör de föreslagna övningarna. När du funderar över hur du ska svara, tänk då på alla de olika övningar/rörelser som du blivit ombedd att göra som en del av din behandling.

1. **Jag gör mina övningar så ofta som jag blivit rekommenderad.**

*Instämmer helt Instämmer inte alls*

Top of Form

| *0*  ☐ | *1*  ☐ | *2*  ☐ | *3*  ☐ | *4*  ☐ |
| --- | --- | --- | --- | --- |

1. **Jag glömmer bort att göra mina övningar.**

*Instämmer helt Instämmer inte alls*

Top of Form

| *0*  ☐ | *1*  ☐ | *2*  ☐ | *3*  ☐ | *4*  ☐ |
| --- | --- | --- | --- | --- |

1. **Jag tränar mindre än vad min behandlande kontakt i sjukvården rekommenderat.**

*Instämmer helt Instämmer inte alls*

Top of Form

| *0*  ☐ | *1*  ☐ | *2*  ☐ | *3*  ☐ | *4*  ☐ |
| --- | --- | --- | --- | --- |

1. **Jag lyckas få in mina övningar i mina dagliga rutiner.**

*Instämmer helt Instämmer inte alls*

Top of Form

| *0*  ☐ | *1*  ☐ | *2*  ☐ | *3*  ☐ | *4*  ☐ |
| --- | --- | --- | --- | --- |

1. **Jag kommer mig inte för att göra mina övningar.**

*Instämmer helt Instämmer inte alls*

Top of Form

| *0*  ☐ | *1*  ☐ | *2*  ☐ | *3*  ☐ | *4*  ☐ |
| --- | --- | --- | --- | --- |

**6. Jag gör de flesta eller alla mina övningar.**

*Instämmer helt Instämmer inte alls*

| *0*  ☐ | *1*  ☐ | *2*  ☐ | *3*  ☐ | *4*  ☐ |
| --- | --- | --- | --- | --- |

**Section B: Exercise Adherence Rating Scale (EARS)**

For each of the following 6 statements, please tick the box which best describes how you do your recommended exercises/activities. When thinking about your answer, please consider any exercises/activities that you have been asked to do as part of your treatment.

1. **I do my exercises as often as recommended**

*Completely agree Completely disagree*

Top of Form

| *0*  ☐ | *1*  ☐ | *2*  ☐ | *3*  ☐ | *4*  ☐ |
| --- | --- | --- | --- | --- |

1. **I forget to do my exercises**

*Completely agree Completely disagree*

Top of Form

| *0*  ☐ | *1*  ☐ | *2*  ☐ | *3*  ☐ | *4*  ☐ |
| --- | --- | --- | --- | --- |

1. **I do less exercise than recommended by my healthcare professional**

*Completely agree Completely disagree*

Top of Form

| *0*  ☐ | *1*  ☐ | *2*  ☐ | *3*  ☐ | *4*  ☐ |
| --- | --- | --- | --- | --- |

**4. I fit my exercises into my regular routine**

*Completely agree Completely disagree*

Top of Form

| *0*  ☐ | *1*  ☐ | *2*  ☐ | *3*  ☐ | *4*  ☐ |
| --- | --- | --- | --- | --- |

**5. I don’t get around to doing my exercises**

*Completely agree Completely disagree*

Top of Form

| *0*  ☐ | *1*  ☐ | *2*  ☐ | *3*  ☐ | *4*  ☐ |
| --- | --- | --- | --- | --- |

**6. I do most, or all, of my exercises**

*Completely agree Completely disagree*

Top of Form

| *0*  ☐ | *1*  ☐ | *2*  ☐ | *3*  ☐ | *4*  ☐ |
| --- | --- | --- | --- | --- |
